# Transcriptional response of *Candida albicans* to *Pseudomonas aeruginosa* in a polymicrobial biofilm

**DOI:** 10.1093/g3journal/jkab042

**Published:** 2021-02-12

**Authors:** Ruan Fourie, Errol D Cason, Jacobus Albertyn, Carolina H Pohl

**Affiliations:** 1 Department of Microbial, Biochemical and Food Biotechnology, University of the Free State, Bloemfontein, 9301, South Africa; 2 Department of Animal Wildlife and Grassland Sciences, University of the Free State, Bloemfontein, 9301, South Africa

**Keywords:** *Candida albicans*, *Pseudomonas aeruginosa*, polymicrobial biofilm, hypoxia, metal homeostasis, morphogenesis

## Abstract

*Candida albicans* is frequently co-isolated with the Gram-negative bacterium, *Pseudomonas aeruginosa*. *In vitro*, the interaction is complex, with both species influencing each other. Not only does the bacterium kill hyphal cells of *C. albicans* through physical interaction, it also affects *C. albicans* biofilm formation and morphogenesis, through various secreted factors and cell wall components. The present study sought to expand the current knowledge regarding the interaction between *C. albicans* and *P. aeruginosa*, using transcriptome analyses of early static biofilms. Under these conditions, a total of 2,537 open reading frames (approximately 40% of the *C. albicans* transcriptome) was differentially regulated in the presence of *P. aeruginosa.* Upon deeper analyses it became evident that the response of *C. albicans* toward *P. aeruginosa* was dominated by a response to hypoxia, and included those associated with stress as well as iron and zinc homeostasis. These conditions may also lead to the observed differential regulation of genes associated with cell membrane synthesis, morphology, biofilm formation and phenotypic switching. Thus, *C. albicans* in polymicrobial biofilms with *P. aeruginosa* have unique transcriptional profiles that may influence commensalism as well as pathogenesis.

## Introduction

Interkingdom interactions are ubiquitous in nature and can affect various aspects of growth, antimicrobial resistance and virulence of species within a consortium (Polke *et al.* 2015). These interactions are frequently encountered in polymicrobial associations formed between the opportunistic fungal pathogen, *Candida albicans*, and commensal microorganisms as well as pathobionts in humans (Morales and Hogan, 2010; Diaz *et al.* 2012; Neville *et al.* 2015). This is, in part, due to the ability to form biofilms on both abiotic and biotic surfaces (Polke *et al.* 2015). This facilitates physical interaction, interaction with various secreted molecules and competition for nutrients (De Sordi and Mühlschlegel 2009; Elias and Banin 2012).

*C. albicans* is frequently co-isolated with the Gram-negative bacterium, *Pseudomonas aeruginosa* (Haiko *et al.* 2019). *In vitro*, the interaction is complex, with both species influencing each other (Fourie and Pohl 2019). The bacterium was found to lyse and kill hyphal cells of *C. albicans* through physical interaction (Hogan and Kolter 2002; Brand *et al.* 2008; Bandara *et al.* 2010). In addition, it affects *C. albicans* biofilm formation and morphogenesis, through various secreted factors and cell wall components (Hogan *et al.* 2004; McAlester *et al.* 2008; Bandara *et al.* 2010, 2013; Holcombe *et al.* 2010). This includes inhibition of morphogenesis from yeast to hyphal morphologies by phenazines, quorum sensing molecules, lipopolysaccharides, and via sequestration of iron, as well as promotion of morphogenesis by peptidoglycan. These stimuli elicit their effects through various signaling pathways in *C. albicans* (Shareck and Belhumeur 2011). Therefore, multiple stimuli, occurring simultaneously, from co-incubation with *P. aeruginosa* may play a role to affect the morphology of *C. albicans*. This research entailed a deeper look into the interaction between *C. albicans* and *P. aeruginosa* in polymicrobial biofilms, using RNAseq, with a focus on the transcriptional response of *C. albicans*.

## Materials and methods

### Strain maintenance

*C. albicans* SC5314 was stored at −80°C with 15% glycerol. Yeast strains were revived and maintained on yeast malt (YM) agar (3 g l^−1^ malt extract, 3 g l^−1^ yeast extract, 5 g l^−1^ peptone, 10 g l^−1^ glucose, 16 g l^−1^ agar) at 30 °C. *P. aeruginosa* PAO1 was stored at −80°C with 25% glycerol and revived and maintained on Luria-Bertani (LB) agar (5 g l^−1^ yeast extract, 10 g l^−1^ tryptone, 10 g l^−1^ sodium chloride, and 15 g l^−1^ agar).

### Mono- and poly-microbial biofilm formation

Formation of biofilms were adapted from previous studies (Ells *et al.* 2011; Fourie *et al.* 2017). For monomicrobial biofilm formation, *C. albicans* was grown on YM agar for 24 hours at 30 °C, inoculated into 10 mL yeast nitrogen base (YNB) broth (10 g l^−1^ glucose, 6.7 g l^−1^ YNB) and incubated at 30 °C for 24 hours. Cells were harvested (1,878 × *g*, 5 minutes) and the supernatant removed. This was followed by washing the cells twice with phosphate buffered saline (PBS) (Oxoid, England). The cells were counted with a hemocytometer and diluted to 1 × 10^6^ cells/mL in 20 mL filter sterilized (0.22 μm nitrocellulose filter, Merck Millipore, Ireland) RPMI-1640 medium (Sigma-Aldrich, USA). Standardized cell suspensions were dispensed into 90 mm polystyrene petri dishes (Merck, Germany), covered with Parafilm^®^ and incubated for 6 hours at 37 °C to allow biofilm formation.

For polymicrobial biofilm formation, *P. aeruginosa* was grown on LB plates for 24 hours at 37 °C. Cells were inoculated into 5 mL nutrient broth (1 g l^−1^ malt extract, 2 g l^−1^ yeast extract, 5 g l^−1^ peptone and 8 g l^−1^ sodium chloride) and incubated at 37 °C for 24 hours with shaking (150 rpm). These cells were washed three times with PBS and diluted to an optical density (OD_600_) of approximately 0.05 in RPMI-1640 medium containing 1 × 10^6^ cells/mL *C. albicans* and biofilms grown as described in above. Both mono- and poly-microbial biofilms were prepared and visualized with scanning electron microscopy as described in Fourie *et al.* (2017).

### Total RNA extraction

Biofilms were scraped off and centrifuged (1,971 × *g*, 3 minutes at 4 °C). A total of five biofilms were pooled per sample. This was done in triplicate for both mono- and poly-microbial biofilms. Supernatant was aspirated from mono- and poly-microbial biofilms and 2 mL RNA*later^®^* (Qiagen) added to prevent RNA degradation before storage at −80°C. Stored biofilms samples were thawed on ice and centrifuged at 4,000 *g* for 5 minutes. RNA*later^®^* was aspirated, and total RNA extraction was carried out using the RNeasy protect mini kit (Qiagen). At the same time, the DNA present was removed with RNase-Free DNase Set (Qiagen), according to the manufacturer’s instructions. The quality of RNA in each sample was determined before sequencing at the Centre for Proteomic and Genomic Research (CPGR, Cape Town, South Africa). Quality tests carried out include checking for contaminants with the use of the NanoDrop ND1000, determining absolute concentration using the Qubit^®^ RNA HS Assay Kit, as well as evaluating the integrity using the Agilent Bioanalyzer Nano Assay. A total of 1 µg of RNA per sample was treated with the Illumina Ribo-Zero rRNA Removal Kit to remove ribosomal RNA. After ribosomal RNA removal, samples were purified (Agencourt RNAClean XP Kit, Beckman Coulter) and indexed libraries were prepared using the ScriptSeq^TM^ v2 RNA-Seq Library Preparation Kit and ScriptSeq^TM^ Index PCR Primers—Set 1 (Illumina). The sizes of the libraries were profiled with the Bioanalyzer High Sensitivity Assay Kit (Agilent) and quantified (Qubit^®^ HS DNA Assay Kit). Samples were diluted and a 1% Phix control library (Illumina) was spiked into samples. Sequencing was completed on the Nextseq 500 (Illumina) using Nextseq 500 High Output (150 cycle) Kit.

### Analysis of differentially expressed genes

The resultant fastq files were analyzed for quality by FastQC (v0.11.5; Andrews 2010) and low-quality reads and bases were discarded using PRINSEQ-lite (v0.20.4; Schmieder and Edwards 2011). *C. albicans* samples (monomicrobial and polymicrobial) were aligned to the *C. albicans* SC5314 genome (assembly 21; The *Candida* Genome Database; Skrzypek *et al.* 2017) via TopHat2 (Trapnell *et al.* 2012; Kim *et al.* 2013) using the fr-secondstrand option, which gave the best overall alignment rate (Dutton *et al.* 2016). Aligned files were merged with SAMtools (Li *et al.* 2009) and the resultant BAM files, constructed with TopHat2, were used to construct gene expression count tables with the use of the BEDTools multicov command (Quinlan and Hall 2010). Differential expression of genes was analyzed and heatmaps and principle component analysis plots were constructed with DESeq2 (Love *et al.* 2014), with modified commands described at “https://gist.github.com/stephenturner/f60c1934405c127f09a6”.

### Determination of overrepresented functional classes of genes and transcription factors

To determine which functional classes of genes are overrepresented in our data sets, PANTHER (Protein Alignment Through Evolutionary Relationship) was utilized (Mi *et al.* 2013; 2016). Furthermore, gene lists of differentially expressed genes were evaluated for overrepresented transcription factors (TFs) using Pathogenic Yeast Search for Transcriptional Regulators And Consensus Tracking (PathoYeastract) (Monteiro *et al.* 2016).

### Confirmation of differential expression with nCounter^®^

Confirmation of differential expression was performed using nCounter^®^ with Elements^TM^ XT Reagents according to manufacturer’s specifications (Geiss *et al.* 2008). A multiplexed probe library (nCounter^®^ elements CodeSet) was designed with two sequence-specific probes for genes of interest. Probes were mixed with approximately 100 ng of purified total RNA and allowed to hybridize (20 hours, 67 °C). Samples were loaded on an nCounter^®^ SPRINT^TM^ Cartridge and processed with an nCounter^®^ SPRINT Profiler (NanoString Technologies, USA). Results were processed in nSolver 4.0 software. This was done for one sample of each condition.

## Results and discussion

### Exploratory analysis of RNAseq data

**Figure 1:**
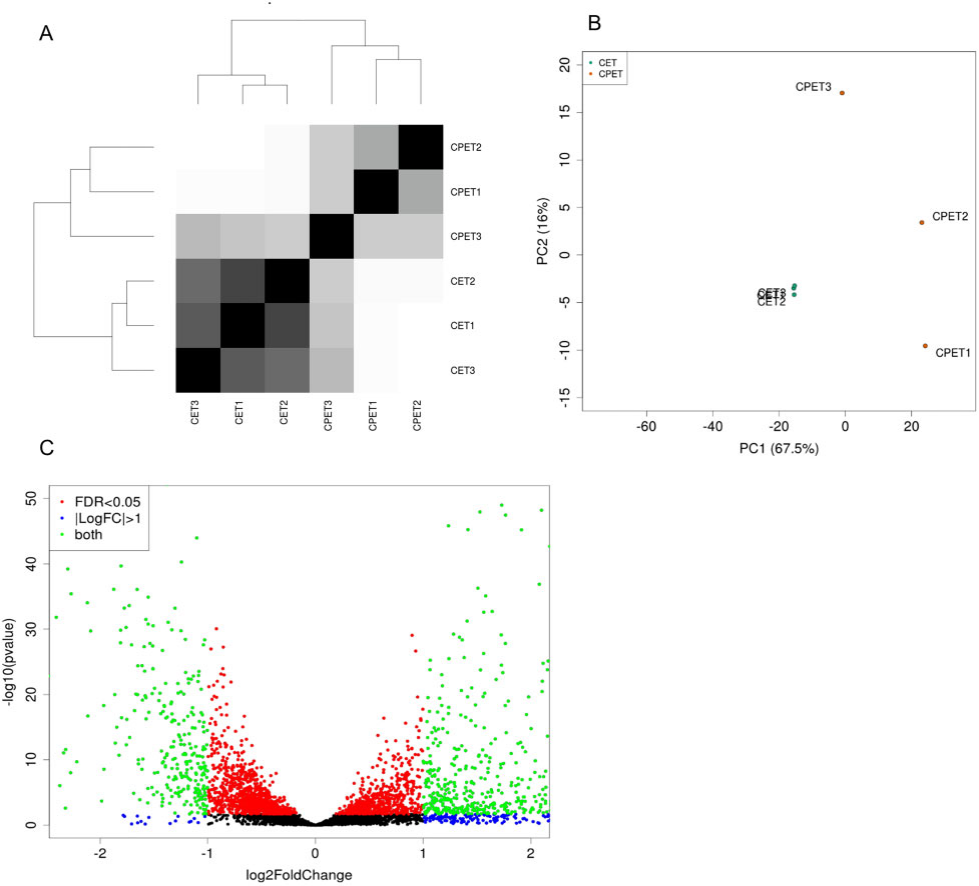
Distance between control and experimental biofilms using sample distance heat maps and principle component analysis plots constructed with DESeq2. (A) sample distance heat map, (B) principle component analysis plot, (C) Volcano plots of significant differentially expressed genes with Log10(*P*-value) of differentially expressed genes on *y*-axis with the *x*-axis representing the log_2_ fold change of these genes. Red dots indicate differential expression with a False Discovery Rate (FDR) of less than 0.05. Blue dots indicate genes that are differentially expressed with a log_2_ fold change of above 1. Green represents genes adhering to both criteria. CPET—polymicrobial biofilms of *C. albicans* and *P. aeruginosa* CET—*C. albicans* monomicrobial biofilms.

Analysis of raw data revealed excellent quality data, with approximately 92.6% of sequences having a Phred-score of above Q30. An average of 4.3% of low-quality sequences were removed, yielding an average of 126.2 × 10^6^ sequences (150 bp paired-end) per sample. These raw reads were aligned to the genome of *C. albicans* SC5314 and further evaluated for differential expression. Complete expression profiles were compared to determine overall differences. As seen in Figure 1A, monomicrobial biofilms of *C. albicans* are very similar in terms of their expression profiles with low distance between replicates, compared to polymicrobial biofilms. *C. albicans* co-cultured with *P. aeruginosa* also cluster together, however, large variation is seen between replicates compared to monomicrobial counterparts. These differences in gene expression profiles are expected due to large variation in population dynamics and microcolony formation in polymicrobial biofilms (Stacy *et al.* 2016). The large difference between mono-and poly-microbial biofilms is apparent through the amount of significantly differentially expressed genes (Figure 1B) with a total of 2,537 open reading frames (ORFs) (with a *p*adj < 0.05) corresponding to approximately 40% of the *C. albicans* transcriptome being altered due to the presence of *P. aeruginosa* (Supplementary Table S1). Furthermore, 917 ORFs are significantly differentially expressed at a log2fold change (L2FC) threshold of above 1 or below −1 in the presence of *P. aeruginosa* (Figure 1C).

Grainha *et al.* (2020) used previously published data to determine the interaction between *C. albicans* and *P. aeruginosa* in polymicrobial biofilms. From these data, it is evident that various different experimental approached have been taken by researchers. When comparing our data with the most relevant high throughput experiments performed previously, we can see that in general there is little overlap between any specific data sets (Supplementary Figure S1), with no genes or proteins shared by all data sets and most being unique to a specific study. The data sets that have the most overlap with our current study is that of Bandara *et al.* (2020) for upregulated genes (22 in total) and Trejo-Hernandez *et al.* (2014) for downregulated proteins (8 in total). This lack of overlap is not surprising as there are significant experimental differences in all these studies.

### Effect of *P. aeruginosa* on *C. albicans* transcriptome in early static biofilms

To determine the effect of the mentioned conditions on the transcriptome of *C. albicans*, an approach was utilized that includes the functional analysis of gene lists to determine overrepresented functional classes of genes. It is necessary to mention that, although the functional annotation of genes is ever expanding, only a portion of *C. albicans* genes are annotated, with many genes with unknown function (as of January 27, 2020, 70.20% or 4,365 of ORFs are still uncharacterized; Skrzypek *et al.* 2017). Thus, the data represented here in terms of gene enrichment analysis do not incorporate many genes currently without GO annotations. Analysis of functional classes of genes that are differentially expressed in response to co-incubation with *P. aeruginosa* is given in Table 1 (repressed genes) and Table 2 (induced genes). For repressed genes below the Log2FC threshold of −1, 79 out of 264 (29.92%) genes were annotated to at least one GO term. For induced genes above the Log2FC threshold of 1,184 of 653 (28.18%) genes were annotated to at least GO term. Genes associated with each GO term can be found in Supplementary Table S3.

**Table 1:** **Overrepresented GO Terms of *C. albicans* repressed genes in response to co-incubation with *P. aeruginosa* in a polymicrobial biofilm.** Over-represented Gene Ontology (GO) terms with PANTHER (Mi *et al.* 2013) of significantly (*p*adj < 0.05) differentially expressed genes with a log_2_ fold change threshold of 1/-1. Significance based on Fisher’s Exact with False Discovery Rate (FDR) multiple test correction. Unclassified indicates genes that could not be attributed to a specific GO term.

GO term	No. of differentially expressed genes	Fold enrichment	*P*-value	FDR
**Cellular localization of products of repressed genes according to GO cellular component**				
Cytosolic small ribosomal subunit (GO : 0022627)	3	24.04	0.000873	0.0157
Mitochondrial respiratory chain complex III (GO : 0005750)	3	24.04	0.000873	0.0153
Proton-transporting ATP synthase complex, catalytic core F(1) (GO : 0045261)	3	19.23	0.00137	0.0221
Proton-transporting ATP synthase complex, coupling factor F(o) (GO : 0045263)	4	12.82	0.000641	0.0118
Hyphal cell wall (GO : 0030446)	16	7.54	2.78E-09	1.07E-07
Cell surface (GO : 0009986)	26	5.02	7.59E-11	4.09E-09
Yeast-form cell wall (GO : 0030445)	8	4.75	0.000506	0.00952
Plasma membrane (GO : 0005886)	41	3.4	1.54E-11	8.91E-10
Extracellular region (GO : 0005576)	21	3.32	3.32E-06	9.94E-05
Cellular component (GO : 0005575)	174	1.26	5.94E-10	2.4E-08
Unclassified	18	0.33	5.94E-10	2.53E-08
**Function of repressed genes according to GO biological process**				
Cellular zinc ion homeostasis (GO : 0006882)	3	32.05	0.00051	0.02
Hydrogen peroxide catabolic process (GO : 0042744)	3	32.05	0.00051	0.0199
ATP synthesis coupled proton transport (GO : 0015986)	8	15.08	4.23E-07	4.1E-05
Tricarboxylic acid cycle (GO : 0006099)	8	12.21	1.51E-06	0.000118
Cellular iron ion homeostasis (GO : 0006879)	6	11.31	4.66E-05	0.00234
Electron transport chain (GO : 0022900)	13	7.71	7.19E-08	8.48E-06
Cellular oxidant detoxification (GO : 0098869)	6	7.69	0.000278	0.0115
Cellular response to reactive oxygen species (GO : 0034614)	5	7.28	0.00113	0.0404
Translation (GO : 0006412)	46	6.55	5.97E-23	2.6E-19
Cell adhesion (GO : 0007155)	9	3.61	0.00139	0.0488
Symbiosis, encompassing mutualism through parasitism (GO : 0044403)	13	3.39	0.000219	0.0092
Pathogenesis (GO : 0009405)	27	2.57	1.13E-05	0.000695
Response to external stimulus (GO : 0009605)	20	2.26	0.000881	0.0318
Filamentous growth of a population of unicellular organisms (GO : 0044182)	28	2.06	0.000345	0.0139
Biological process (GO : 0008150)	182	1.16	3.66E-07	3.63E-05
Unclassified	10	0.29	3.66E-07	3.71E-05

**Table 2:** **Overrepresented GO Terms of *C. albicans* induced genes in response to co-incubation with *P. aeruginosa* in a polymicrobial biofilm.** Overrepresented Gene Ontology (GO) terms with PANTHER (Mi *et al.* 2013) of significantly (*p*adj < 0.05) differentially expressed genes with a log_2_ fold change threshold of 1/-1. Significance based on Fisher’s Exact with False Discovery Rate (FDR) multiple test correction. Unclassified indicates genes that could not be attributed to a specific GO term.

GO term	No. of differentially expressed genes	Fold enrichment	*P*-value	FDR
**Cellular localization of products of repressed genes according to GO cellular component**				
Membrane raft (GO : 0045121)	6	10.76	8.6E-05	0.0058
Anchored component of membrane (GO : 0031225)	23	5.05	2.12E-09	4.28E-07
Cell surface (GO : 0009986)	31	4.02	4.41E-10	1.19E-07
Extracellular region (GO : 0005576)	35	3.71	2.09E-10	8.44E-08
Fungal-type cell wall (GO : 0009277)	21	3.2	8.29E-06	0.000745
Plasma membrane (GO : 0005886)	40	2.22	4.6E-06	0.000465
Macromolecular complex (GO : 0032991)	20	0.47	7.33E-05	0.00539
**Function of repressed genes according to GO biological process**				
Xenobiotic transport (GO : 0042908)	4	21.51	0.000232	0.0361
Organic hydroxy compound transport (GO : 0015850)	6	10.76	8.6E-05	0.0156
Drug export (GO : 0046618)	5	10.76	0.000347	0.0421
Lipid catabolic process (GO : 0016042)	9	6.45	3.88E-05	0.00995
Fatty acid biosynthetic process (GO : 0006633)	7	6.27	0.000332	0.0414
Alcohol biosynthetic process (GO : 0046165)	8	5.55	0.000254	0.0357
Adhesion of symbiont to host (GO : 0044406)	10	4.58	0.000172	0.0279
Cellular response to oxidative stress (GO : 0034599)	14	3.76	5.97E-05	0.0124
Pathogenesis (GO : 0009405)	45	2.87	8.65E-10	1.26E-06
Biofilm formation (GO : 0042710)	17	2.79	0.000276	0.0365
Carbohydrate metabolic process (GO : 0005975)	19	2.59	0.000296	0.0379
Cellular response to drug (GO : 0035690)	28	2.08	0.000415	0.0476
Oxidation-reduction process (GO : 0055114)	40	2.04	3.23E-05	0.0101
Biological process (GO : 0008150)	258	1.1	0.000238	0.0358
Unclassified	28	0.54	0.000238	0.0346

### Confirmation of RNA-Seq data with NanoString

**Figure 2:**
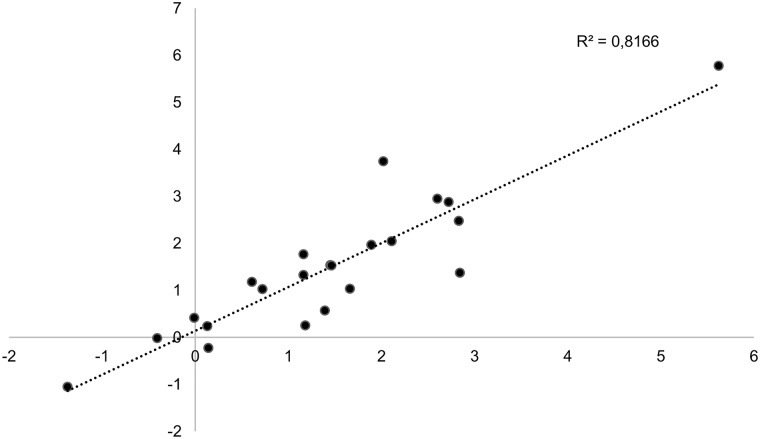
Correlation between Log2fold change values through Nanostring compared to RNA-Seq. Scatter plot of the Log2fold change values obtained through RNA-Seq and Nanostring of genes of interest (Supplementary Table S2) indicating the correlation between these two methods (*R*^2^ = 0.817).

The fold change obtained using RNA-Seq compared with that from NanoString, using selected genes of interest is shown in Supplementary Table S2. The similarity between the transcript levels obtained from the two platforms indicates consistency in the data generated for the analysis. Figure 2 indicates the linear correlation between RNA-Seq compared to Nanostring concerning the genes in Supplementary Table S2 with a correlation coefficient (*r*) of 0.90 and *R*^2^ of 0.82.

### Response to low-oxygen levels

In our data set, genes involved in ATP synthesis, tricarboxylic acid cycle as well as electron transport chain are repressed. Interestingly, induction of genes related to carbohydrate metabolic process is seen. This response is expected during fermentative growth (Askew *et al.* 2009). In addition, several genes involved in ethanol production (*ADH5*, *ADH3*, orf19.4504, and *ADH2*) are induced in our data set (Supplementary Table S1). The repression of the tricarboxylic acid cycle and electron transport chain, together with the induction of the carbohydrate metabolic process and alcohol biosynthetic process may indicate that *C. albicans* relies more on fermentation for energy acquisition during co-incubation with *P. aeruginosa*.

Interestingly, genes involved in glycolysis, fermentation, stress response, cell wall, fatty acid, iron metabolism and hyphae specific genes are induced during hypoxia, whilst the tricarboxylic acid cycle, respiration and ATP synthesis are repressed (Askew *et al.* 2009). This response is similar to what is observed here in polymicrobial biofilms of *C. albicans* and *P. aeruginosa*, suggesting that the response of *C. albicans* toward *P. aeruginosa* may be dominated by the response toward hypoxia. The carbohydrate metabolism of *C. albicans* in a hypoxic environment is regulated by Tye7p and Gal4p (Askew *et al.* 2009; Bonhomme *et al.* 2011). Confirming our observation, is the induction of *TYE7* (Table 3) as well as *GAL4* (Supplementary Table S1), the former of which is needed for growth and virulence in a hypoxic environment.

Aerobic metabolism by *P. aeruginosa* in polymicrobial biofilms with *C. albicans* may quickly deplete available oxygen in the biofilm, forcing hypoxia quicker than in *C. albicans* monomicrobial biofilms. In addition to induced hypoxia, inhibition of *C. albicans* metabolic activity due to the production of the redox-active phenazine compounds by *P. aeruginosa*, has been reported previously (Morales *et al.* 2013). Morales *et al.* (2013) reported an increase in fermentation products, such as ethanol, by *C. albicans*, due to the action of these phenazine compounds. Considering this, the observed effect on *C. albicans* carbohydrate metabolism may be due to not only hypoxia, but also phenazine production by *P. aeruginosa*. Interestingly, ethanol decreases *P. aeruginosa* motility and stimulates *P. aeruginosa* biofilm formation and phenazine production (Chen *et al.* 2014; Lewis *et al.* 2019).

### Stress responses

GO biological process reveals a response toward stress (*e.g.*, chemical) with response to xenobiotic as well as drug and organic hydroxy compound transport being overrepresented. This response may be due to toxic phenazines produced by *P. aeruginosa*. In addition, the cellular response to oxidative stress is overrepresented in induced genes (Table 2). The glutathione-dependent S-nitrosoglutathione reductase (encoded by *FDH3*), the nitric oxide dioxygenase (encoded by *YHB1*) as well as the transcriptional regulator (encoded by *CTA4*), playing a role in nitrosative radical detoxification, is induced in our data set (Supplementary Table S1). This may indicate a response to nitrosative stress of *C. albicans* in the presence of *P. aeruginosa* (Ullmann *et al.* 2004; Chiranand *et al.* 2008; Tillmann *et al.* 2015). Nitrosative radicals may be from both endogenous and exogenous origin, such as the use of nitrite as an alternative electron acceptor as well as from the redox-active phenazines produced by *P. aeruginosa* (Chiranand *et al.* 2008). Although the gene encoding for catalase, *CAT1*, playing a role in oxidative stress response, is repressed in our data set, the genes encoding for superoxide dismutase (SOD) *SOD1* and *SOD6* are significantly induced (Supplementary Table S1). SODs protect cells against reactive oxygen species generated by the mitochondrial respiratory chain and external sources such as the oxidative burst during phagocytosis. *SOD1* has been linked to virulence (Martchenko *et al.* 2004), while the function of *SOD6* still needs to be elucidated (Frohner *et al.* 2009). Interestingly, the mitochondrial SOD, encoded by *SOD2*, is not differentially regulated, indicating that the possible increase in oxidative stress is localized in the cytosol, and not due to mitochondrial activity. This may indicate that the source of oxidative stress is not due to cellular activity, but due to external factors, such as phenazines, entering the cell.

### Membrane formation and organization

Lipid catabolic process, including lipase genes (*LIP1—LIP3*, *LIP6*, *LIP8—LIP10*), catalyzing hydrolysis or synthesis of triacyclglycerols (Schofield *et al.* 2005; Gácser *et al.* 2007) is overrepresented in induced genes (Table 2). In addition, fatty acid biosynthetic process is induced, possibly to counteract physical damage to *C. albicans* plasma membrane by *P. aeruginosa* (Brand *et al.* 2008). Importantly, several TFs, with roles in membrane and cell wall synthesis, such as *UPC2*, *STP4*, and *SUT1* are overrepresented in our data set and are significantly differentially expressed (Supplementary Table S1). In addition, Upc2 is associated with the regulation of a hypoxic response, inducing genes involved in ergosterol biosynthesis (MacPherson *et al.* 2005; Synnott *et al.* 2010) and Sut1p is involved in sterol uptake during hypoxia in *S. cerevsiae* (Foster *et al.* 2013). Interestingly, Sut1p is also involved in zinc acquisition in *C. albicans* (Xu *et al.* 2015).

### Iron and zinc homeostasis

Previous research suggests that *C. albicans* in combination with *P. aeruginosa* elicits an iron-deprivation response in *C. albicans* due to rapid iron-sequestration by *P. aeruginosa* siderophores (Singh *et al.* 2011; Purschke *et al.* 2012; Trejo-Hernández *et al.* 2014). This iron-deficient response is mediated by a Cap2-HAP complex, affecting various genes including three iron uptake pathways and repression of iron utilization and storage (Singh *et al.* 2011). However, closer examination of our data revealed only a modest response toward iron deprivation in terms of repression of iron utilization genes [including *ACO1* and *IDH2* (involved in aerobic respiration), *QCR2* (respiratory electron transport chain) and haem containing proteins, like *CAT1* (hydrogen peroxide detoxification)], indicating a possible reduced dependency on iron (Table 1). This repression of iron usage genes is similar to the results obtained by Trejo-Hernández *et al.* (2014) in a proteomic study of monomicrobial and polymicrobial biofilms. None of the transcription factors (*SFU1*, *SEF1*, or *HAP43*) involved in iron regulation (Chen *et al.* 2011; Singh *et al.* 2011) was differentially regulated at 6 hours of co-incubation.

Strikingly, several genes involved in iron uptake and usage is repressed in our data set, resembling an abundance of iron compared to monomicrobial biofilms. These genes include components of the reductive iron pathway, namely those that encode the high-affinity iron permease *FTR1*, the multicopper ferroxidase *FET34*, the ferric/cupric reductase *CFL2*, the ferritin receptor *ALS3* and the heme oxygenase *HMX1*. This may indicate that less iron is required for cellular growth when *C. albicans* is co-incubated with *P. aeruginosa*, possibly due to growth repression. This contrasts with what has been reported before (Trejo-Hernández *et al.* 2014), although this may be due to differences in incubation conditions and duration of biofilm formation.

Genes involved in zinc acquisition are repressed in our data set (Supplementary Table S1), including components of a “zincophore” system, namely, the genes encoding zinc transporters (*ZRT1*, *ZRT2*, and *ZRT3*) as well as the extracellular zinc binding protein *PRA1*, responsible for zinc sequestration (Böttcher *et al.* 2015; Łoboda and Rowińska-Źyrek 2017). This “zincophore” system is under positive regulation of Csr1p (also known as Zap1p). *CSR1* is repressed in our data set, indicating that *C. albicans* is under less zinc-limitation during co-incubation with *P. aeruginosa*, similar to the effect on iron, discussed above.

### Morphogenesis and biofilm formation

**Figure 3:**
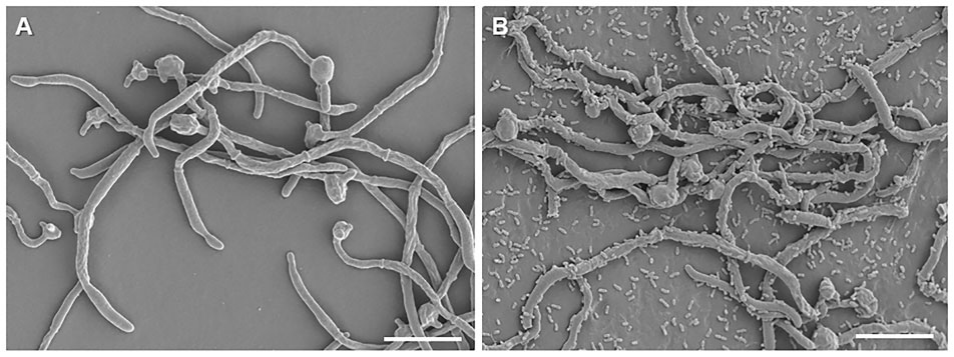
Scanning electron micrograph of monomicrobial and polymicrobial biofilms. (A) *C. albicans* monomicrobial biofilm, grown for 6 hours, indicating attached hyphae, (B) Polymicrobial biofilm, grown for 6 hours, indicating the attachment of *P. aeruginosa* to *C. albicans* hyphae. Scale bar represents 10 µm.

Scanning electron micrographs (Figure 3) indicate the attached *C. albicans* hyphae in monomicrobial and polymicrobial biofilms after 6 hours of incubation. In the polymicrobial biofilms, *P. aeruginosa* attached to the hyphae can be seen. Previous research found that *C. albicans* morphogenesis is inhibited in the presence of *P. aeruginosa* due to the N-acyl-homoserine lactone (AHL), 3-oxo-homoserine lactone, produced by the bacterium (Hogan *et al.* 2004). *C. albicans* yeast and pseudohyphal morphologies are characterized by a morphology dependent set of expressed genes. Similarly, true hyphae formation is transcriptionally unique (Bensen *et al.* 2002) and include *HWP1*, *ECE1*, *HYR1*, *RBT1*, *RBT4*, *RBT5*, and *WAP1*, which encode GPI-modified cell wall proteins. A number of these genes, including *HWP1* and *ECE1*, are repressed in the presence of *P. aeruginosa* in our data set, associated with a predominant yeast and pseudohyphal transcriptional profile (Supplementary Table S1). In contrast, *HYR1* and *CHT2*, associated with a true hyphal-morphology, is induced in our data set.

Adhesion is a crucial component of the process of biofilm formation of *C. albicans*. In our data set, genes associated with adhesion is repressed in the presence of *P. aeruginosa* (Table 1). In addition, several TFs with roles in adhesion, including *ZFU2*, *AHR1*, and *TRY4*, are differentially regulated (Table 3). *ZFU2* and *TRY4* are TFs that regulate yeast form adherence (Böhm *et al.* 2017). However, *ZFU2* is repressed, whereas *TRY4* is induced with contrasting effects. In addition, Ahr1p recruits Mcm1p and induce initial surface adhesion during biofilm formation (Askew *et al.* 2011). Interestingly, both *AHR1* and *MCM1* are repressed in our data set. In addition, the yeast wall protein *YWP1*, whose expression negatively correlates with adhesion (Granger *et al.* 2005), is induced in response to *P. aeruginosa*, further providing evidence for an effect on adhesion.

A number of external stimuli, that may influence filamentation and biofilm formation, is found in the presence of *P. aeruginosa* (Fourie *et al.* 2016). These stimuli include hypoxia (induces filamentation), presence of LPS (represses filamentation), cell wall and membrane stress (induces filamentation), phenazines (repress filamentation) and quorum sensing molecules. It is tempting to speculate that these many stimuli may present as an exaggerated response that could be detrimental to adaptation to a host environment. Therefore, in addition to TFs, chromatin-remodeling has been shown to affect biofilm formation and filamentation through integrating signals from external stimuli and acting as a transcriptional buffer affecting transcription kinetics (Hnisz *et al.* 2010, 2012; Kim *et al.* 2012; Garnaud *et al.* 2016). The Set3/Hos2 complex (Set3C) is a NAD-dependent histone deacetylation complex [consisting of four core subunits (Set3p, Hos2p, Snt1p, and Sif2p) and three peripheral subunits (Hos4p, Hst1p, and Cpr1p) (Hnisz *et al.* 2010)] that binds directly to most of the master regulators of biofilm formation and is involved in the dispersal of cells from mature biofilms, via the modulation of *NRG1* expression levels (Nobile *et al.* 2014). *SET3* as well as the gene encoding the peripheral subunit, *HST1*, is significantly induced in our data set. This raises the question if Set3C may play a role in modulating filamentation of *C. albicans* in the presence of *P. aeruginosa*, possibly inducing the dispersion of *C. albicans*, to escape the hostile activity of *P. aeruginosa* during co-incubation.

### Phenotypic switching

In addition to affecting biofilm formation in *C. albicans*, the Set3C is also a regulator of white-opaque switching (Hnisz *et al.* 2010; Garnaud *et al.* 2016). Strikingly, the gene encoding the white-opaque regulator, *WOR1*, is induced in our data set (Supplementary Table S1). This may indicate that white-opaque switching could take place. However, *WOR2* expression (required for stabilization of the opaque phenotype) is significantly repressed (Table 3), indicating that switching may be unstable and transient. In addition, whereas *WH11* and *EFG1* expression is associated with white-phase and *OP4* and *SAP1* expression is associated with the opaque phase (Tao *et al.* 2014), contradicting results are obtained in this data set, with both *WH11* and *OP4* being induced. In addition to white and opaque phenotypes, a gray phenotype can also manifest, however, comparison with expression results obtained by Tao *et al.* (2014) revealed little overlap between our data set and opaque or gray specific transcriptional responses. The overexpression of *WOR1* in the presence of bacteria has been reported before and is required for commensal fitness (Fox *et al.* 2014). In these cases the full opaque program was also not induced and the authors speculate that additional signals may be required. Ramírez-Zavala *et al.* (2008) showed that switching could be induced in *C. albicans* by an anaerobic environment in the presence of high temperature (37 °C), however, this only occurred in the WO-1 and CAI-4 strains, and could not be replicated in the SC5314 strain that is heterozygous at the MTL(a/α). Due to this, the strain of *C. albicans* (SC5314) may experience an induction of *WOR1* in the presence of *P. aeruginosa* due to hypoxia, however, the full opaque program may not be induced as a trisomy of chromosome 1 and homozygosity of the mating type locus is needed. It is however interesting to note that an increase in expression of genes encoding hydrolytic enzymes, such as *SAP3* and lipases (*LIP1* to *LIP3*, *LIP6*, *LIP8* to *LIP10*), associated with the opaque phenotype (Lan *et al.* 2002), is seen in our data set. This provides evidence that the opaque expression profile, at least in part, may be induced by co-incubation of *C. albicans* with *P. aeruginosa*.

## Conclusions

For the formation of both mono- and poly-microbial biofilms, a stationary model adapted from Fourie *et al.* (2017), was followed, which allows for the rapid depletion of oxygen and formation of microaerophilic to hypoxic biofilms, similar to the biofilms formed in the lungs of cystic fibrosis patients (Crocker *et al.* 2019). The formation of microaerophilic to hypoxic biofilms may be a more accurate representation of *in vivo* conditions, compared to planktonic growth of cells with aeration, as was previously used to determine the effect of *P. aeruginosa* on *C. albicans* (Holcombe *et al.* 2010). In addition, the present model also does not include a distinct adhesion phase as in many other biofilm studies. In our opinion, this also may more closely reflects *in vivo* conditions, as biofilms involved in infection do not undergo such an initial adhesion phase and may therefore be more complex. Under these conditions, co-incubation of *C. albicans* with *P. aeruginosa* exhibited a strong response toward hypoxia, with a shift toward fermentative growth and sterol metabolism. Furthermore, the hypoxic environment may have driven the reduced expression of genes associated with iron and zinc acquisition, as hypoxia may decrease the need for these metals. Interestingly, *SET3* transcription is upregulated in the presence of *P. aeruginosa*, indicating that the Set3C may be contributing to the interaction of *C. albicans* with *P. aeruginosa*, by acting as a transcriptional buffer to control yeast filamentation and dispersal. Furthermore, the white-opaque regulator, *WOR1*, is upregulated in the presence of *P. aeruginosa*, indicating that switching to the opaque-phenotype may be taking place, although additional signals may be required to initiate the full opaque-transcriptional profile. From these data, it is clear that these polymicrobial biofilms have a unique transcriptional profile, indicating several shifts in metabolism that may play important roles in commensalism as well as infection.

### Data availability

Supplementary files for this investigation are available at figshare (https://doi.org/10.25387/g3.13791607). Supplementary Table S1 comprises the raw analyzed data from RNASeq experiments. Supplementary Table S2 shows a comparison between the transcripts obtained using RNA-Seq and NanoString. Supplementary Table S3 indicates the genes associated with each identified GO term. All raw data for this investigation have been deposited in the NCBI’s GEO database under the accession number GSE136726.

## Funding

This work was supported by the National Research Foundation of South Africa (grant number 115566 and 118543 to CHP). The funders played no role in the study or in the preparation of the article or decision to publish. 

*Conflict of interest*: All authors declare that they have no conflict of interest.

## References

[jkab042-B1] Andrews S. 2010. FastQC a quality-control tool for high-throughput sequence data. Bioinformatics doi:citeulike-article-id:11583827

[jkab042-B2] Askew C , Sellam A, Epp E, Hogues H, Mullick A, et al 2009. Transcriptional regulation of carbohydrate metabolism in the human pathogen *Candida albicans*. PLoS Pathog. 5:e1000612. 19816560 10.1371/journal.ppat.1000612 PMC2749448

[jkab042-B3] Askew C , Sellam A, Epp E, Mallick J, Hogues H, et al 2011. The zinc cluster transcription factor Ahr1p directs Mcm1p regulation of *Candida albicans* adhesion. Mol. Microbiol. 79:940–953. 21299649 10.1111/j.1365-2958.2010.07504.x PMC4092010

[jkab042-B4] Bandara HMHN , Cheung BP, Watt RM, Jin LJ, Samaranayake LP. 2013. *Pseudomonas aeruginosa* lipopolysaccharide inhibits *Candida albicans* hyphae formation and alters gene expression during biofilm development. Mol Oral Microbiol. 28:54–69. 23194472 10.1111/omi.12006

[jkab042-B5] Bandara HMHN , Yau JYY, Watt RM, Jin LJ, Samaranayake LP. 2010. *Pseudomonas aeruginosa* inhibits *in-vitro Candida* biofilm development. BMC Microbiol. 10:125. 20416106 10.1186/1471-2180-10-125 PMC2874548

[jkab042-B6] Bandara HMHN , Wood DLA, Vanwonterghem I, Hugenholtz P, Cheung BPK, et al 2020. Fluconazole resistance in *Candida albicans* is induced by *Pseudomonas aeruginosa* quorum sensing. Sci Rep. 10:7769. 32385378 10.1038/s41598-020-64761-3 PMC7211000

[jkab042-B7] Bensen ES , Filler SG, Berman J. 2002. A forkhead transcription factor is important for true hyphal as well as yeast morphogenesis in *Candida albicans*. Eukaryot Cell 1:787–798. 12455696 10.1128/EC.1.5.787-798.2002 PMC126749

[jkab042-B8] Böhm L , Torsin S, Tint SH, Eckstein MT, Ludwig T, et al 2017. The yeast form in the fungus *Candida albicans* promotes persistence in the gut of gnotobiotic mice. PLoS Pathog. 13:e1006699. 29069103 10.1371/journal.ppat.1006699 PMC5673237

[jkab042-B9] Bonhomme J , Chauvel M, Goyard S, Roux P, Rossignol T, et al 2011. Contribution of the glycolytic flux and hypoxia adaptation to efficient biofilm formation by *Candida albicans*. Mol Microbiol. 80:995–1013. 21414038 10.1111/j.1365-2958.2011.07626.x

[jkab042-B10] Böttcher B , Palige K, Jacobsen ID, Hube B, Brunke S. 2015. *Csr1/Zap1* maintains zinc homeostasis and influences virulence in *Candida dubliniensis* but is not coupled to morphogenesis. Eukaryot Cell 14:661–670. 26002718 10.1128/EC.00078-15 PMC4486669

[jkab042-B11] Brand A , Barnes JD, Mackenzie KS, Odds FC, Gow NAR. 2008. Cell wall glycans and soluble factors determine the interaction between the hyphae of *Candida albicans* and *Pseudomonas aeruginosa*. FEMS Microbiol Lett. 287:48–55. 18680523 10.1111/j.1574-6968.2008.01301.x PMC2613227

[jkab042-B12] Chen AI , Dolben EF, Okegbe C, Harty CE, Golub Y, et al 2014. *Candida albicans* ethanol stimulates *Pseudomonas aeruginosa* WspR-controlled biofilm formation as part of a cyclic relationship involving phenazines. PLoS Pathog. 10:e1004480. 25340349 10.1371/journal.ppat.1004480 PMC4207824

[jkab042-B13] Chen C , Pande K, French SD, Tuch BB, Noble SM. 2011. An iron homeostasis regulatory circuit with reciprocal roles in *Candida albicans* commensalism and pathogenesis. Cell Host Microbe 10:118–135. 21843869 10.1016/j.chom.2011.07.005 PMC3165008

[jkab042-B14] Chiranand W , McLeod I, Zhou H, Lynn JJ, Vega LA, et al 2008. *CTA4* transcription factor mediates induction of nitrosative stress response in *Candida albicans*. Eukaryot Cell 7:268–278. 18083829 10.1128/EC.00240-07 PMC2238162

[jkab042-B15] Crocker AW , Harty CE, Hammond JH, Willger SD, Salazar P, et al 2019. *Pseudomonas aeruginosa* ethanol oxidation by AdhA in low-oxygenenvironments. J Bacteriol. 201:e00393-19. [10.1128/JB.00393-19] 31527114 10.1128/JB.00393-19 PMC6832066

[jkab042-B16] De Sordi L , Mühlschlegel FA. 2009. Quorum sensing and fungal-bacterial interactions in *Candida albicans*: a communicative network regulating microbial coexistence and virulence. FEMS Yeast Res. 9:990–999. 19845041 10.1111/j.1567-1364.2009.00573.x

[jkab042-B17] Diaz PI , Xie Z, Sobue T, Thompson A, Biyikoglu B, et al 2012. Synergistic interaction between *Candida albicans* and commensal oral Streptococci in a novel *in vitro* mucosal model. Infect Immun 80:620–632. 22104105 10.1128/IAI.05896-11 PMC3264323

[jkab042-B18] Dutton LC , Paszkiewicz KH, Silverman RJ, Splatt PR, Shaw S, et al 2016. Transcriptional landscape of trans-kingdom communication between *Candida albicans* and *Streptococcus gordonii*. Mol Oral Microbiol. 31:136–161. 26042999 10.1111/omi.12111 PMC4670286

[jkab042-B20] Elias S , Banin E. 2012. Multi-species biofilms: living with friendly neighbors. FEMS Microbiol Rev. 36:990–1004. 22229800 10.1111/j.1574-6976.2012.00325.x

[jkab042-B21] Ells R , Kock JLF, Albertyn J, Kemp G, Pohl CH. 2011. Effect of inhibitors of arachidonic acid metabolism on prostaglandin E_2_ production by *Candida albicans* and *Candida dubliniensis* biofilms. Med Microbiol Immunol. 200:23–28. 20821232 10.1007/s00430-010-0169-7

[jkab042-B22] Fourie R , Ells R, Kemp G, Sebolai OM, Albertyn J, et al 2017. *Pseudomonas aeruginosa* produces aspirin insensitive eicosanoids and contributes to the eicosanoid profile of polymicrobial biofilms with *Candida albicans*. Prostaglandins Leukot Essent Fatty Acids 117:36–46. 28237086 10.1016/j.plefa.2017.01.008

[jkab042-B23] Fourie R , Ells R, Swart CW, Sebolai OM, Albertyn J, et al 2016. *Candida albicans* and *Pseudomonas aeruginosa* interaction, with focus on the role of eicosanoids. Front Physiol. 7:64. 26955357 10.3389/fphys.2016.00064 PMC4767902

[jkab042-B24] Fourie R , Pohl CH. 2019. Beyond antagonism: the interaction between *Candida* species and *Pseudomonas aeruginosa*. J Fungi 5:34. 10.3390/jof5020034 PMC6617365 31010211

[jkab042-B25] Foster HA , Cui M, Naveenathayalan A, Unden H, Schwanbeck R, et al 2013. The zinc cluster protein Sut1 contributes to filamentation in *Saccharomyces cerevisiae*. Eukaryot Cell 12:244–253. 23223039 10.1128/EC.00214-12 PMC3571311

[jkab042-B26] Fox EP , Cowley ES, Nobile CJ, Hartooni N, Newman DK, et al 2014. Anaerobic bacteria grow within *Candida albicans* biofilms and induce biofilm formation in suspension cultures. Curr Biol. 24:2411–2416. 25308076 10.1016/j.cub.2014.08.057 PMC4252622

[jkab042-B27] Frohner IE , Bourgeois C, Yatsyk K, Majer O, Kuchler K. 2009. *Candida albicans* cell surface superoxide dismutases degrade host-derived reactive oxygen species to escape innate immune surveillance. Mol Microbiol. 71:240–252. 19019164 10.1111/j.1365-2958.2008.06528.x PMC2713856

[jkab042-B28] Gácser A , Stehr F, KröGer C, Kredics L, SchäFer W, et al 2007. Lipase 8 affects the pathogenesis of *Candida albicans*. Infect Immun. 75:4710–4718. 17646357 10.1128/IAI.00372-07 PMC2044512

[jkab042-B29] Garnaud C , Champleboux M, Maubon D, Cornet M, Govin J. 2016. Histone deacetylases and their inhibition in *Candida* species. Front Microbiol. 7:1238. 27547205 10.3389/fmicb.2016.01238 PMC4974301

[jkab042-B30] Geiss GK , Bumgarner RE, Birditt B, Dahl T, Dowidar N, et al 2008. Direct multiplexed measurement of gene expression with color-coded probe pairs. Nat Biotechnol. 26:317–325. 18278033 10.1038/nbt1385

[jkab042-B31] Grainha T , Jorge P, Alves D, Lopes SP, Pereira MA. 2020. Unraveling *Pseudomonas aeruginosa* and *Candida albicans* communication in coinfection scenarios: insights through network analysis. Front Cell Infect Microbiol. 10:550505. 33262953 10.3389/fcimb.2020.550505 PMC7686562

[jkab042-B32] Granger BL , Flenniken ML, Davis DA, Mitchell AP, Cutler LE. 2005. Yeast wall protein 1 of *Candida albicans*. Microbiology 151:1631–1644. 15870471 10.1099/mic.0.27663-0

[jkab042-B33] Haiko J , Saeedi B, Bagger G, Karpati F, Özenci V. 2019. Coexistence of *Candida* species and bacteria in patients with cystic fibrosis. Eur J Clin Microbiol Infect Dis. 38:1071–1077. 30739228 10.1007/s10096-019-03493-3 PMC6520323

[jkab042-B34] Hnisz D , Bardet AF, Nobile CJ, Petryshyn A, Glaser W, et al 2012. A histone deacetylase adjusts transcription kinetics at coding sequences during *Candida albicans* morphogenesis. PLoS Genet. 8:e1003118. 23236295 10.1371/journal.pgen.1003118 PMC3516536

[jkab042-B35] Hnisz D , Majer O, Frohner I, Komnenovic EV, Kuchler K. 2010. The Set3/Hos2 histone deacetylase complex attenuates cAMP/PKA signalling to regulate morphogenesis and virulence of *Candida albicans*. PLoS Pathog. 6:e1000889. 20485517 10.1371/journal.ppat.1000889 PMC2869326

[jkab042-B75687265] Hogan DA. 2002. Pseudomonas-Candida Interactions: An Ecological Role for Virulence Factors. Science. 296:2229–2232. 12077418 10.1126/science.1070784

[jkab042-B36] Hogan DA , Vik A, Kolter R. 2004. A *Pseudomonas aeruginosa* quorum-sensing molecule influences *Candida albicans* morphology. Mol Microbiol. 54:1212–1223. 15554963 10.1111/j.1365-2958.2004.04349.x

[jkab042-B37] Holcombe LJ , McAlester G, Munro CA, Enjalbert B, Brown AJ, et al 2010. *Pseudomonas aeruginosa* secreted factors impair biofilm development in *Candida albicans*. Microbiology 156:1476–1486. 20150241 10.1099/mic.0.037549-0

[jkab042-B38] Kim D , Pertea G, Trapnell C, Pimentel H, Kelley R, et al 2013. TopHat2: accurate alignment of transcriptomes in the presence of insertions, deletions and gene fusions. Genome Biol. 14:R36. 23618408 10.1186/gb-2013-14-4-r36 PMC4053844

[jkab042-B39] Kim T , Xu Z, Clauder-Münster S, Steinmetz LM, Buratowski S. 2012. Set3 HDAC mediates effects of overlapping non-coding transcription on gene induction kinetics. Cell 150:1158–1169. 22959268 10.1016/j.cell.2012.08.016 PMC3461055

[jkab042-B40] Lan C-Y , Newport G, Murillo LA, Jones T, Scherer S, et al 2002. Metabolic specialization associated with phenotypic switching in *Candida albicans*. Proc Natl Acad Sci USA. 99:14907–14912. 12397174 10.1073/pnas.232566499 PMC137518

[jkab042-B41] Lewis KA , Baker AE, Chen AI, Harty CE, Kuchma SL. 2019. Ethanol decreases *Pseudomonas aeruginosa* flagellar motility through the regulation of flagellar stators. J Bacteriol. 201:e00285-19. [10.1128/JB.00285-19] 31109994 10.1128/JB.00285-19 PMC6707923

[jkab042-B42] Li H , Handsaker B, Wysoker A, Fennell T, Ruan J, et al 2009. The sequence alignment/map format and SAMtools. Bioinformatics 25:2078–2079. 19505943 10.1093/bioinformatics/btp352 PMC2723002

[jkab042-B43] Łoboda D , Rowińska-Żyrek M. 2017. Zinc binding sites in Pra1, a zincophore from *Candida albicans*. Dalton Trans. 46:13695–13703. 28725901 10.1039/c7dt01675a

[jkab042-B44] Love MI , Huber W, Anders S. 2014. Moderated estimation of fold change and dispersion for RNAseq data with DESeq2. Genome Biol. 15:550. 25516281 10.1186/s13059-014-0550-8 PMC4302049

[jkab042-B45] MacPherson S , Akache B, Weber S, De Deken X, Raymond M, et al 2005. *Candida albicans* zinc cluster protein Upc2p confers resistance to antifungal drugs and is an activator of ergosterol biosynthetic genes. Antimicrob Agents Chemother. 1745–1752. 49:1745–1752. 15855491 10.1128/AAC.49.5.1745-1752.2005 PMC1087678

[jkab042-B46] Martchenko M , Alarco A-M, Harcus D, Whiteway M. 2004. Superoxide dismutases in *Candida albicans*: transcriptional regulation and functional characterization of the hyphal-induced *SOD5* gene. Mol Biol Cell 15:456–467. 14617819 10.1091/mbc.E03-03-0179 PMC329211

[jkab042-B47] McAlester G , O'Gara F, Morrissey JP. 2008. Signal-mediated interactions between *Pseudomonas aeruginosa* and *Candida albicans*. J Med Microbiol. 57:563–569. 18436588 10.1099/jmm.0.47705-0

[jkab042-B48] Mi H , Huang X, Muruganujan A, Tang H, Mills C. 2016. PANTHER version 11: expanded annotation data from Gene Ontology and Reactome pathways, and data analysis tool enhancements. Nucleic Acids Res. 45:D183–D189. [10.1093/nar/gkw1138] 27899595 10.1093/nar/gkw1138 PMC5210595

[jkab042-B49] Mi H , Muruganujan A, Thomas PD. 2013. PANTHER in 2013: modeling the evolution of gene function, and other gene attributes, in the context of phylogenetic trees. Nucleic Acids Res. 41:D377–D386. 23193289 10.1093/nar/gks1118 PMC3531194

[jkab042-B50] Monteiro PT , Pais P, Costa C, Manna S, Sá-Correia I, et al 2016. The PathoYeastract database: an information system for the analysis of gene and genomic transcription regulation in pathogenic yeasts. Nucleic Acids Res. 45:D597–D603. 27625390 10.1093/nar/gkw817 PMC5210609

[jkab042-B51] Morales DK , Grahl N, Okegbe C, Dietrich LEP, Jacobs N, et al 2013. Control of *Candida albicans* metabolism and biofilm form ation by *Pseudomonas aeruginosa* phenazines. mBio 4:e00526-12. 23362320 10.1128/mBio.00526-12 PMC3560528

[jkab042-B52] Morales DK , Hogan DA. 2010. *Candida albicans* interactions with bacteria in the context of human health and disease. PLoS Pathog. 6:e1000886. 20442787 10.1371/journal.ppat.1000886 PMC2861711

[jkab042-B53] Neville BA , d'Enfert C, Bougnoux M-E. 2015. *Candida albicans* commensalism in the gastrointestinal tract. FEMS Yeast Res. 15:fov081. 26347504 10.1093/femsyr/fov081

[jkab042-B54] Nobile CJ , Fox EP, Hartooni N, Mitchell KF, Hnisz D, et al 2014. A histone deacetylase complex mediates biofilm dispersal and drug resistance in *Candida albicans*. mBio 5:e01201-14. 24917598 10.1128/mBio.01201-14 PMC4056552

[jkab042-B55] Polke M , Hube B, Jacobsen ID. 2015. *Candida* survival strategies. Adv Appl Microbiol. 91:139–235. 25911234 10.1016/bs.aambs.2014.12.002

[jkab042-B56] Purschke FG , Hiller E, Trick I, Rupp S. 2012. Flexible survival strategies of *Pseudomonas aeruginosa* in biofilms result in increased fitness compared with *Candida albicans*. Mol Cell Proteomics. 11:1652–1669. [10.1074/mcp.M112.017673] 22942357 10.1074/mcp.M112.017673 PMC3518115

[jkab042-B57] Quinlan AR , Hall IM. 2010. BEDTools: a flexible suite of utilities for comparing genomic features. Bioinformatics 26:841–842. 20110278 10.1093/bioinformatics/btq033 PMC2832824

[jkab042-B58] Ramírez-Zavala B , Reuß O, Park Y-N, Ohlsen K, Morschhäuser J. 2008. Environmental induction of white-opaque switching in *Candida albicans*. PLoS Pathog. 4:e1000089. 18551173 10.1371/journal.ppat.1000089 PMC2405950

[jkab042-B59] Schmieder R , Edwards R. 2011. Quality control and preprocessing of metagenomic datasets. Bioinformatics 27:863–864. 21278185 10.1093/bioinformatics/btr026 PMC3051327

[jkab042-B60] Schofield DA , Westwater C, Warner T, Balish E. 2005. Differential *Candida albicans* lipase gene expression during alimentary tract colonization and infection. FEMS Microbiol Lett. 244:359–365. 15766791 10.1016/j.femsle.2005.02.015

[jkab042-B61] Shareck J , Belhumeur P. 2011. Modulation of morphogenesis in *Candida albicans* by various small molecules. Eukaryot Cell 10:1004–1012. 21642508 10.1128/EC.05030-11 PMC3165445

[jkab042-B62] Singh RP , Prasad HK, Sinha I, Agarwal N, Natarajan K. 2011. Cap2-HAP complex is a critical transcriptional regulator that has dual but contrasting roles in regulation of iron homeostasis in *Candida albicans*. J Biol Chem. 286:25154–25170. 21592964 10.1074/jbc.M111.233569 PMC3137088

[jkab042-B63] Skrzypek MS , Binkley J, Binkley G, Miyasato SR, Simison M, et al 2017. The *Candida* Genome Database (CGD): incorporation of assembly 22, systematic identifiers and visualization of high throughput sequencing data. Nucleic Acids Res. 45:D592–D596. 27738138 10.1093/nar/gkw924 PMC5210628

[jkab042-B64] Stacy A , McNally L, Darch SE, Brown SP, Whiteley M. 2016. The biogeography of polymicrobial infection. Nat Rev Microbiol. 14:93–105. 26714431 10.1038/nrmicro.2015.8 PMC5116812

[jkab042-B65] Synnott JM , Guida A, Mulhern-Haughey S, Higgins DG, Butler G. 2010. Regulation of the hypoxic response in *Candida albicans*. Eukaryot Cell 9:1734–1746. 20870877 10.1128/EC.00159-10 PMC2976306

[jkab042-B66] Tao L , Du H, Guan G, Dai Y, Nobile CJ, et al 2014. Discovery of a “white-gray-opaque” tristable phenotypic switching system in *Candida albicans*: roles of non-genetic diversity in host adaptation. PLoS Biol. 12:e1001830. 24691005 10.1371/journal.pbio.1001830 PMC3972085

[jkab042-B67] Tillmann AT , Strijbis K, Cameron G, Radmaneshfar E, Thiel M, et al 2015. Contribution of Fdh3 and Glr1 to glutathione redox state, stress adaptation and virulence in *Candida albicans*. PLoS ONE 10:e0126940. 26039593 10.1371/journal.pone.0126940 PMC4454436

[jkab042-B68] Trapnell C , Roberts A, Goff L, Pertea G, Kim D, et al 2012. Differential gene and transcript expression analysis of RNA-seq experiments with TopHat and Cufflinks. Nat Protoc. 7:562–579. 22383036 10.1038/nprot.2012.016 PMC3334321

[jkab042-B69] Trejo-Hernández A , Andrade-Domínguez A, Hernández M, Encarnación S. 2014. Interspecies competition triggers virulence and mutability in *Candida albicans*–*Pseudomonas aeruginosa* mixed biofilms. ISME J. 8:1974–1988. 24739628 10.1038/ismej.2014.53 PMC4184018

[jkab042-B70] Ullmann BD , Myers H, Chiranand W, Lazzell AL, Zhao Q, et al 2004. Inducible defense mechanism against nitric oxide in *Candida albicans*. Eukaryot Cell 3:715–723. 15189992 10.1128/EC.3.3.715-723.2004 PMC420131

[jkab042-B72] Xu W , Solis NV, Ehrlich RL, Woolford CA, Filler SG, et al 2015. Activation and alliance of regulatory pathways in *C. albicans* during mammalian infection. PLoS Biol. 13:e1002076. 25693184 10.1371/journal.pbio.1002076 PMC4333574

